# Cell-Free DNA Fragmentation Patterns as Biomarkers for Human Papillomavirus-Related Cancers: A Systematic Review of Methodological Diversity and Diagnostic Performance

**DOI:** 10.3390/ijms27146372

**Published:** 2026-07-17

**Authors:** Fernanda Santos, Marta S. Silva, Francisco A. Caramelo, Magda M. Santana, Rui J. Nobre, Jorge M. P. Tomaz, Luís P. Almeida, Margarida Figueiredo-Dias

**Affiliations:** 1 Faculty of Medicine, Gynecology Department, University of Coimbra, 3004-504 Coimbra, Portugal; marg.fig.dias@gmail.com; 2 Coimbra Academic and Clinical Centre, 3000-075 Coimbra, Portugal; 3 Gynecology Department, ULS—Coimbra Hospital and University Centre of Coimbra, 3004-561 Coimbra, Portugal; 4 Gene and Stem Cell Therapies for the Brain Group, Center for Neuroscience and Cell Biology (CNC), 3004-504 Coimbra, Portugal; martasilva@cnc.uc.pt (M.S.S.); magdamsantana@gmail.com (M.M.S.); rui.jorge.nobre@gmail.com (R.J.N.); luispa@cnc.uc.pt (L.P.A.); 5 Vectors, Gene and Cell Therapy Group, Center for Innovative Biomedicine and Biotechnology (CIBB), 3004-504 Coimbra, Portugal; 6 GeneT, Center for Excellence in Gene Therapy in Portugal, University of Coimbra, 3004-504 Coimbra, Portugal; 7 Laboratory of Biostatistics and Medical Informatics (LBIM), Faculty of Medicine, University of Coimbra, 3000-548 Coimbra, Portugal; fcaramelo@fmed.uc.pt; 8 Centre for Innovative Biomedicine and Biotechnology (CIBB), University of Coimbra, 3004-504 Coimbra, Portugal; 9 Institute for Interdisciplinary Research (IIIUC), University of Coimbra, 3030-789 Coimbra, Portugal; 10 Blood and Transfusion Medicine Department, ULS—Coimbra Hospital and University Centre of Coimbra, 3004-561 Coimbra, Portugal; jtomaz7@gmail.com

**Keywords:** cell-free DNA, fragmentomics, HPV-related cancers, TTMV-HPV DNA, diagnostic performance

## Abstract

Novel blood biomarkers are crucial for HPV-related cancers to overcome the limitations of imaging and biopsies. This review evaluates the diagnostic performance of circulating cell-free DNA (cfDNA) fragmentomic patterns, distinguishing host-genome integrity from specific viral signatures. Following PRISMA guidelines, we searched PubMed, EMBASE, and Web of Science up to February 2026. Methodological quality was assessed via QUADAS-2, and diagnostic performance was synthesized using a random-effects model. From 489 records, six studies (215 patients, 209 controls) met inclusion criteria. Lacking host-derived fragmentomics data, the analysis focused exclusively on the structural size profiles of circulating viral DNA (cfHPV-DNA). Meta-analysis of four cohorts specifically evaluating these viral fragmentation patterns yielded a pooled Diagnostic Odds Ratio (DOR) of 205.73 (95% CI: 44.72–946.38). Specificity was robust (~100%), with high sensitivity (>90%) for macroscopic disease. However, sensitivity decreased in low-tumor-burden cohorts. cfHPV-DNA fragmentation patterns shows promising diagnostic potential for macroscopic HPV-driven malignancies. However, further studies must determine whether fragment sizing truly outperforms binary viral detection and correlates with disease severity. Furthermore, while current assays exploit the analytical simplicity of viral targets, host-derived fragmentomics remains an overlooked compartment that warrants exploration to determine its true clinical value regarding underlying tumor dynamics. Systematic Review Registration: PROSPERO, identifier: CRD420251052768.

## 1. Introduction

Human Papillomavirus (HPV) is a major oncogenic driver, responsible for a substantial global cancer burden, accounting for 831,204 new cancer cases and 422,935 deaths worldwide in 2022. Despite the availability of highly effective primary and secondary prevention measures, most of these cases (88.6%) and deaths (89.6%) occur in women, with cervical cancer accounting for 75.6% [1]. Persistent infection with high-risk HPV underlies virtually all cervical cancers and contributes significantly to anal (88%), vaginal (70%), vulvar (43%), penile (50%), and oropharyngeal (26–70%) cancers [1,2]. Consequently, the aggregate burden of these HPV-driven diseases is substantial, with annual direct medical costs in the United States exceeding $9 billion. Notably, this expenditure is largely driven by the cumulative costs of cervical cancer screening, diagnosis, and management [3].

Despite ongoing advances in healthcare, the diagnosis and longitudinal surveillance of HPV-associated malignancies remain predominantly dependent on invasive tissue biopsies and radiologic imaging. However, both modalities exhibit limitations regarding early-stage detection, minimal residual disease, and subclinical recurrence [4]. This diagnostic challenge is exacerbated for HPV-driven cancers for which no validated screening programs exist [5]. Moreover, the utility of tissue sampling is constrained by its static nature, as a single-site biopsy often fails to recapitulate the entire genomic landscape, misrepresenting intrinsic intratumoral heterogeneity [6]. Addressing these diagnostic challenges, liquid biopsy has emerged as a non-invasive alternative, analyzing tumor-derived components released into various bodily fluids. While applicable to diverse biological matrices, peripheral blood remains the most widely used and clinically informative source. It acts as a central reservoir for a variety of tumor-associated analytes, including circulating tumor cells (CTCs), circulating tumor DNA (ctDNA), tumor-derived extracellular vesicles (EVs), tumor-educated platelets (TEPs), and circulating free RNA (cfRNA). Within this spectrum, ctDNA represents a tumor-specific signal embedded in the background of physiological cell-free DNA (cfDNA), serving as a dynamic indicator that captures the unique genetic and epigenetic landscape of the malignancy [6]. In HPV-associated cancers, this differentiation between signal and background holds unique diagnostic potential. Specifically, the tumor-derived fraction comprises two biologically distinct yet interrelated components: tumor-derived host DNA harboring somatic alterations and circulating viral HPV DNA originating from transcriptionally active viral genomes [7]. Conceptually, this dual molecular origin creates a composite liquid biopsy signal; however, the relative diagnostic contribution of each compartment remains incompletely defined, as clinical evidence has predominantly focused on viral-derived cfDNA.

Nevertheless, conventional liquid biopsy metrics remain inherently limited. Total cfDNA concentration lacks tumor specificity, as it is frequently confounded by underlying inflammatory or physiological states [8]. Conversely, mutation-based assays often fail in early-stage disease due to the scarcity of targetable circulating variants [9]. To overcome these sensitivity barriers, the field has increasingly pivoted toward evaluating the intrinsic physical properties of cfDNA molecules. This paradigm shift has established fragmentomics as a superior biomarker strategy, characterized by the multidimensional analysis of cfDNA physical characteristics, including fragment size topology, specific end-motifs, jagged ends, and nucleosome positioning footprints [10]. Crucially, this multidimensional profiling enables the simultaneous interrogation of two distinct genomic compartments: the specific nucleosomal cleavage patterns of the viral genome and the epigenetic-driven fragmentation signature of the host [11].

However, despite this growing interest, the application of fragmentomics to HPV-driven malignancies remains an under-investigated area characterized by a scarcity of dedicated studies. The current research landscape is highly heterogeneous, varying significantly in methodology (e.g., PCR vs. NGS), target selection (viral vs. host genome), and pre-analytical conditions [8,12]. To date, the diagnostic accuracy of these fragmentation patterns compared to standard diagnostic approaches has not been systematically synthesized, particularly in the context of gynecologic HPV-driven malignancies.

Throughout this review, the term fragmentomics refers to the analysis of structural characteristics of cfDNA fragments, including fragment size distributions and related physical properties. However, when discussing targeted assays such as NavDx, we specifically use the terms fragmentation patterns or fragment size-based detection strategies, as these approaches infer fragment size characteristics rather than performing comprehensive fragmentomic profiling.

Therefore, the main objective of this systematic review and meta-analysis is to evaluate the diagnostic performance of cfDNA fragmentation patterns in detecting HPV-induced cancers. Specifically, we aim to evaluate the current evidentiary landscape regarding differences between viral and host genome fragmentation profiles, while exploring how methodological diversity impacts overall diagnostic accuracy.

## 2. Materials and Methods

### 2.1. Protocol and Registration

This systematic review and meta-analysis adhered to Preferred Reporting Items for Systematic Reviews and Meta-Analyses (PRISMA) 2020 guidelines [13]. The completed PRISMA 2020 Checklist is provided in the Supplementary Materials (Table S1). The review was prospectively registered in PROSPERO (CRD420251052768), and the full protocol is available in the Supporting Information (Table S2).

### 2.2. Eligibility Criteria

Eligible studies included original or replication peer-reviewed research, available in English, Portuguese, or Spanish, without publication year restrictions. In accordance with PROSPERO, eligibility followed an adapted PECO framework for diagnostic test accuracy (DTA) reviews: Exposure = index test (cfDNA fragmentomic assays); Comparators = control groups (healthy volunteers or benign conditions); and Outcomes = diagnostic performance metrics. Specific criteria are detailed in Table S3. Due to the novelty of the field, no sample size restrictions were applied.

### 2.3. Information Sources

We searched PubMed, EMBASE, CENTRAL, Web of Science Core Collection, and major clinical trial registries (WHO, EU, Clinicaltrials.gov), from inception to February 2026. The search combined database-specific syntaxes and keywords covering three blocks: (1) HPV-related malignancies/precursors, (2) liquid biopsy/cfDNA, and (3) cfDNA biophysical features/fragmentomics (Table S2). Grey literature was captured by screening annual conference abstracts (ASCO, ESMO, IPVC) and utilizing Semantic Scholar. Reference lists of included studies and relevant reviews were manually screened, and forward citation tracking was performed. For multiple publications of the same study, the most relevant or largest dataset was selected.

### 2.4. Study Selection and Data Extraction

The systematic literature search was conducted to include all available records published up to 9 February 2026. Duplicates were removed using EndNote version 21.5. Two independent reviewers (F.S., M.S.S.) screened titles, abstracts, and full texts. Disagreements were resolved by consensus or senior reviewers (M.F.-D., R.N.). Extracted variables included: (1) Study characteristics (Author, year, country, funding, design); (2) Clinical data (Sample size, cancer type, HPV status, early/advanced stage stratification [International Federation of Gynecology and Obstetrics (FIGO)/American Joint Committee on Cancer (AJCC)] (3) Pre-analytical/technical details (Matrix, collection/processing, extraction, detection method, fragmentomic metrics); and (4) Diagnostic outcomes (Reference standard, sensitivity, specificity, AUC, raw 2 × 2 data).

### 2.5. Risk of Bias in Individual Studies

Two independent reviewers (F.S., M.F.-D.) evaluated methodological quality using QUADAS-2 [14], resolving discrepancies via a third reviewer (F.C.). Overall evidence certainty was appraised using the GRADE framework adapted for DTA meta-analyses [15].

### 2.6. Statistical Analysis

Meta-analyses were performed using the metafor package in R (v4.1.2). A random-effects model with the restricted maximum-likelihood (REML) estimator was employed. Given the anticipated sparsity of eligible cohorts in this research field, we opted against hierarchical summary receiver operating characteristic (HSROC) modelling. HSROC and bivariate random-effects models may fail to converge or yield unstable parameter estimates when only a few studies are available and sparse 2 × 2 tables contain zero cells [16]. Accordingly, the diagnostic odds ratio (DOR), which combines sensitivity and specificity into a single prevalence-independent measure of diagnostic discrimination [17], was selected as the summary metric to evaluate diagnostic performance given the constraints of the available dataset.

Assessment of publication bias using Deeks’ funnel plot asymmetry test was not performed, as recommended by Cochrane guidance, because fewer than 10 studies were included, below the threshold at which such tests have adequate power to differentiate asymmetry from random variation [18].

## 3. Results

### 3.1. Study Selection

**Figure 1:**
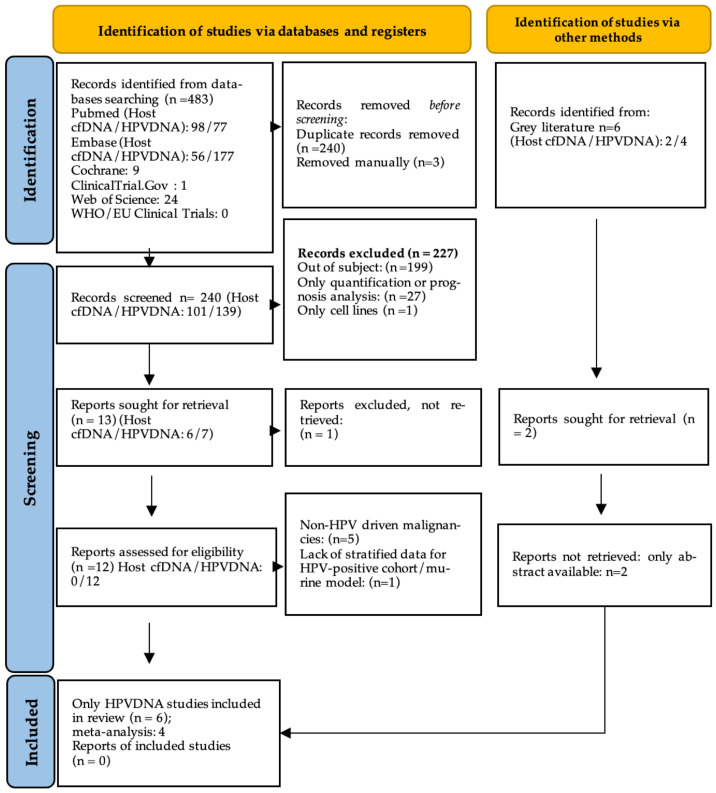
Flowchart describing the study selection process [13].

The initial literature search yielded 489 records (483 from electronic databases and 6 from grey literature). Regarding the database records, after removing 243 duplicates, 240 unique database citations underwent title and abstract screening, resulting in 227 exclusions. Of the 13 reports sought for full-text retrieval, 12 were successfully assessed for eligibility (one record was unretrievable). Six of these were subsequently excluded, primarily for addressing non-HPV malignancies or lacking stratified data. Notably, no host-derived fragmentomics studies provided diagnostic data specific to HPV-driven malignancies. In parallel, screening of the six grey literature records identified two reports sought for retrieval; however, both were unretrievable as only abstracts existed. Ultimately, six cfHPV-DNA studies met the inclusion criteria for qualitative synthesis (Figure 1). Of these six, four provided data suitable for quantitative meta-analysis. Two studies were excluded from quantitative pooling to preserve clinical homogeneity and methodological rigor. First, Gunning et al. [19] was retained qualitatively to establish assay analytical validity, but it lacked a consecutive diagnostic clinical cohort. Second, Rettig et al. [20] analyzed archived pre-diagnostic plasma (collected 19–43 months prior to clinical manifestation). Although highly valuable for demonstrating the potential of fragmentomics in early detection and screening, including this asymptomatic cohort would introduce substantial time-lag bias and artificially underestimate the assay’s true cross-sectional diagnostic accuracy.

### 3.2. Study Characteristics

Since no investigations of host-derived fragmentomics satisfied the inclusion criteria, the following synthesis is exclusively dedicated to the diagnostic performance and biological fragmentation characteristics of circulating viral DNA (cfHPV-DNA), explicitly shifting the focus from conventional viral load quantification to structural fragmentation pattern analysis. Overall, this systematic review included six studies comprising 215 cancer patients (5 with cervical cancer and 210 with Head and Neck Squamous Cell Carcinoma- HNSCC). Of these 215 patients, 205 (including all 5 cervical cancer cases) were included in the quantitative meta-analysis. The control cohorts encompassed 209 individuals (109 included in the meta-analysis), consisting of 155 healthy volunteers (36 with “high-risk” symptoms), 17 with HPV negative- Oropharyngeal Squamous Cell Carcinoma (OPSCC) and one with HPV 18-OPSCC (Table 1 and Table 2). Most studies included patients with OPSCC, and only one study reported two additional cases of non-OPSCC HNSCC [20]. Demographically, among the studies reporting age, the samples consisted predominantly of older adults, generally in their sixth decade of life [20,21,22].

While this review aimed to capture variability in biospecimen types, plasma served as the predominant liquid biopsy matrix across all included diagnostic cohorts. Alternative biofluids were investigated solely by Bhambhani et al. [23], who evaluated matched urine samples alongside plasma. Methodologically, the landscape was dominated by digital droplet PCR (ddPCR) platforms, specifically using the Tumor Tissue Modified Viral (TTMV)-HPV DNA assay [NavDx^®^ (Naveris, Waltham, MA, USA)]. This platform applies an algorithmic fragment size-based detection strategy to discriminate tumor-derived, highly fragmented HPV DNA from longer non-tumor viral sequences, ensuring that detection relies on the distinct physical size of the fragments rather than mere viral presence. This assay was utilized in most clinical cohorts [20,22,24]. Conversely, next-generation sequencing (NGS) strategies, such as the HPV-seq assay used by Leung et al., leverage hybrid-capture sequencing of the full viral genome to comprehensively map the precise fragment size distribution of the viral target, rather than relying on limited amplicons [25]. Detailed technical specifications of the included assays are provided in Supplementary Table S4.

**Table 1:** Baseline clinical and demographic characteristics of the included cfHPV-DNA fragmentomics studies.

Study (Author/ Year)	Country	Study Type	Cancer Type	HPV Status	Sample Size (Total)	Cases (*n*)	Early Stage (*n*)	Advanced Stage (*n*)	Control Group Type	Control (*n*)	Median Age (years/IQR)	Meta-analysis
Gunning et al., 2023 [19]	USA (Naveris, Waltham, MA)	Analytical Validation	HPV + OPSCC	HPV (16, 18, 31, 33, 35)	20,398 (stability)/18 (LOB)	N/A (Analytical)	N/A	N/A	No Template Controls	N/A	N/A	No
Bhambhani et al., 2024 [23]	USA (University of Michigan)	Case–Control	HPV + OPSCC	HPV 16	44	32	0	32	Mixed ^a^	12	NR	Yes
Batool et al., 2023 [21]	USA (Harvard Medical School, Boston)	Prospective Cohort	HPV + OPSCC	HPV (16, 18, 31, 33, 35)	39	3	3	0	‘High-risk’ ^b^ symptoms	36	60 (48–71)	Yes
Ferrandino et al., 2023 [22]	USA (Icahn School of Medicine at Mount Sinai, NY)	Retrospective Observational Cohort	OPSCC	HPV (16, 18, 31, 33, 35)	163	152	T0-T3 ^3^ [*n* = 146] and N0-N0-N2 [*n* = 158]	T4 [*n* = 15] and N3 [*n* = 4]	HPV negative OPSCC	11	63 (56–68.5)	Yes
Leung et al., 2021 [25]	Canada (Princess Margaret Cancer Centre)	Case–control	Cervical cancer and OPSCC	HPV (16, 35, 45, 52, 33 and other types)	68	18 ^c^	NR	NR	Healthy	50	NR	Yes
Rettig et al., 2022 [20]	USA (Harvard Medical School, Boston, MA)	Case–control study (Biobank)	HNSCC	HPV 16	110	10	2	8	Healthy	100	68.5	No

HNSCC: Head and Neck Squamous Cell Carcinoma; IQR: interquartile range; LOB: limit of the blank; NR: not reported; N/A: not applicable; OPSCC: Oropharyngeal Squamous Cell Carcinoma; ^a^ 5 Healthy + 6 HPV negative HNSCC + 1 HPV18 OPSCC; ^b^ ‘High-risk’ _symptoms: Neck mass present for >2 weeks with no signs/symptoms of infection, or with signs/symptoms of infection that did not resolve with antibiotic therapy; Palatine or lingual tonsillar asymmetry detected on physical exam or imaging or Unexplained throat pain for >2 weeks. ^c^ Based on AJCC 8th edition tumor classification, data extracted from Table 1 of [22], minor discrepancies in totals (*n* = 161/162 vs. 163) are due to missing staging data in the original report. ^3^ 5 Cervix + 13 Oropharynx.

**Table 2:** Methodological characteristics and main diagnostic findings of the included cfHPV-DNA fragmentomics studies.

Study (Author/ Year)	DNA Extraction	Detection Method	Viral Target	Fragment Size Logic	Sensitivity (%)	Specificity (%)	TP	TN	FP	FN	Key Findings
Gunning et al., 2023 [19]	QIAamp Circulating Nucleic Acid Kit (Qiagen^®^, Hilden, Germany)	ddPCR (NavDx^®^ Naveris, Waltham, MA, USA) [26,27]	Viral-Fragmentomics ^1^ (via Differential Amplicon Occupancy: Single vs. Double Positive Droplets)	<170 bp (70–170)	N/A ^2^	N/A ^2^	N/A ^2^	N/A ^2^	N/A ^2^	N/A ^2^	NavDx^®^ Naveris, Waltham, MA, USA, shows high analytical sensitivity (LOD~1 cp/uL) and linearity (R2 = 1) for TTMV-HPV.
Bhambhani et al., 2024 [23]	Urine: Q Sepharose resin-based/Plasma: QIAamp-Qiagen^®,^ Hilden, Germany (Standard)	Ultrashort ddPCR (CHAMP-16 Assay, University of Michigan, Ann Arbor, MI, USA)	HPV16 (E6 gene)	<50 bp (urine)/<77 bp (plasma)	90.6	100	29	12	0	3	High concordance <50 bp fragments in urine/plasma.
Batool et al., 2023 [21]	QIAamp Circulating Nucleic Acid Kit (Qiagen^®^, Hilden, Germany)	ddPCR (NavDx^®^ Naveris, Waltham, MA, USA)	TTMV ^1^	<170 bp (70–170)	66.7	100	2	36	0	1	Feasible diagnostic adjunct for high-risk patients. Negative results do not exclude disease.
Ferrandino et al., 2023 [22]	QIAamp Circulating Nucleic Acid Kit (Qiagen^®^, Hilden, Germany)	ddPCR (NavDx^®^ Naveris, Waltham, MA, USA)	TTMV ^1^	<170 bp (70–170)	91.5	100	139	11	0	13	Robust ‘rule-in’ tool for HPV-OPSCC; however, negative results do not exclude malignancy.
Leung et al., 2021 [25]	QIAamp Circulating Nucleic Acid Kit (Qiagen^®^, Hilden, Germany)	Targeted NGS (Illumina NextSeq/ NovaSeq, San Diego, CA, USA)	Full-length Viral Genome	Viral Median~146 bp vs. Host Median~167 bp	100	88	18	44	6	0	Detection of all baseline cases; lowered specificity by identifying latent viral signals in asymptomatic controls.
Rettig et al., 2022 [20]	QIAamp Circulating Nucleic Acid Kit (Qiagen^®^, Hilden, Germany) [24]	ddPCR (NavDx^®^ Naveris, Waltham, MA, USA)	TTMV ^1^	<170 bp (70–170)	30	100	3 ^3^	100	0	7	ctHPV16DNA is detectable in plasma up to 43 months prior to clinical diagnosis.

ddPCR: Droplet Digital PCR; LOD: Limit of detection; NGS: Next-Generation Sequencing; N/A: Not applicable; TTMV-HPV DNA: Tumor-Tissue Modified Viral HPV DNA ^1^: Tumor-Tissue Modified Viral HPV DNA. Note: NavDx® (Naveris, Waltham, MA, USA) uses 12 DNA biomarkers to profile HPV DNA fragmentation via Differential Amplicon Occupancy (DAO), which constitutes the underlying analytical logic for the TTMV-HPV score utilized in the clinical studies. HPV16 is assessed first, with other high-risk HPV types (18, 31, 33, and 35) assayed only if HPV16 is negative; ^2^: Analytical validation study using engineered samples; therefore, clinical diagnostic performance metrics are not applicable; ^3^: ctHPV16DNA was detected in plasma samples from 3 of 10 patients with HPV16-positive HNSCCs (30%, 95% CI = 7–65%), including three of seven patients with HPV16-positive OPSCC (43%, 95% CI = 10–82%), detected 19, 34, and 43 months prior to diagnosis. This cohort was excluded from quantitative pooling to avoid time-lag bias.

### 3.3. Results of Syntheses for Diagnosis

**Figure 2:**
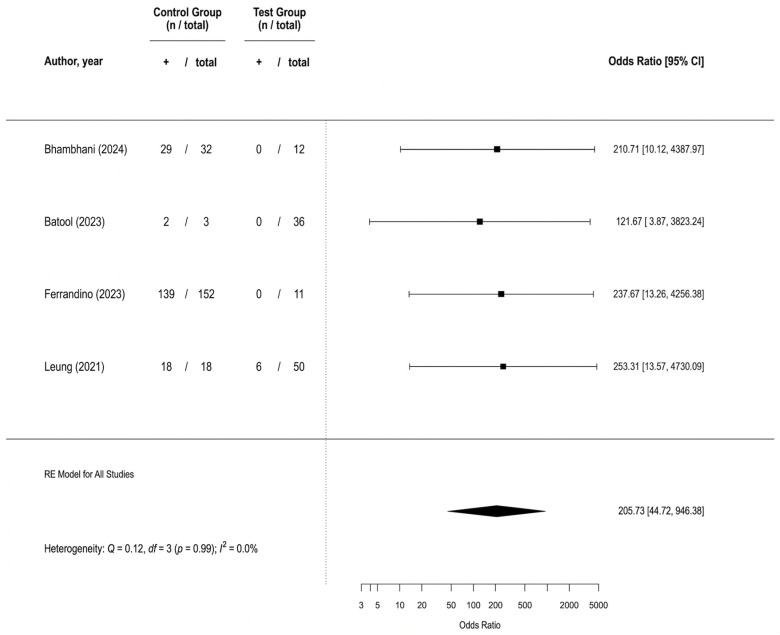
Forest plot of the diagnostic odds ratio (DOR) for assays evaluating cfHPV-DNA fragmentation patterns across the included studies. Individual study DORs and their corresponding 95% confidence intervals (CIs) are depicted by black squares and horizontal lines, respectively. The overall pooled DOR, estimated using a random-effects model, is represented by the black diamond at the bottom. The summary DOR is 205.73 (95% CI: 44.72–946.38), demonstrating high diagnostic accuracy for distinguishing patients with HPV-driven malignancies from control subjects. Statistical heterogeneity was virtually nonexistent (Cochran’s Q = 0.12, df = 3, *p* = 0.99; I^2^ = 0.0%), confirming highly consistent diagnostic discrimination across all evaluated clinical cohorts. The studies included in this analysis are Bhambhani et al. [23], Batool et al. [21], Ferrandino et al. [22], and Leung et al. [25].

The quantitative synthesis of the four eligible studies showed a high overall diagnostic performance. Given the anticipated sparsity of data, HSROC modelling was not performed to avoid model instability and overfitting. Instead, overall diagnostic accuracy was assessed using the DOR. The random-effects meta-analysis yielded a pooled DOR of 205.73 (95% CI: 44.72–946.38) (Figure 2). Statistical heterogeneity across the included cohorts was virtually non-existent (I^2^ = 0.0%; Q = 0.12, *p* = 0.99), indicating highly consistent diagnostic discrimination despite variations in clinical settings (Figure 2).

Beyond these pooled metrics, the qualitative synthesis revealed that methodological heterogeneity across studies directly impacted diagnostic performance and fragmentomic profiling. Notably, while evaluating alternative biofluids, Bhambhani et al. [23] demonstrated that although urine testing is feasible, its diagnostic concordance with plasma is strictly dependent on the selective detection of ultra-short transrenal fragments (<50 bp), which are typically missed by conventional analytical assays. Furthermore, platform selection influenced absolute sensitivity limits. While the widely adopted TTMV-DNA NavDx® (Naveris, Waltham, MA, USA) platform demonstrated robust clinical utility and stability across multiple cohorts by algorithmically profiling these short viral targets [21,22], the high-resolution NGS approach (HPV-seq) achieved 100% diagnostic sensitivity in its respective cohort [25]. This validates the potential of full-genome hybrid-capture NGS to overcome the detection limits of amplicon-based PCR, particularly in low-burden disease [25]. Finally, while diagnostic sensitivity is robust in symptomatic patients, Rettig et al. explored the assay’s performance in the pre-clinical setting using archived biobank samples [20]. They detected the highly fragmented circulating TTMV-HPV DNA signature in 30% (3/10) of patients with HPV16-positive HNSCC, between 19 and 43 months prior to clinical diagnosis. Crucially, specificity remained 100% (0/100) in matched healthy controls, confirming that this fragmentomics-based approach rarely generates false positives even in screening-like scenarios [20].

### 3.4. Reporting Biases

**Figure 3:**
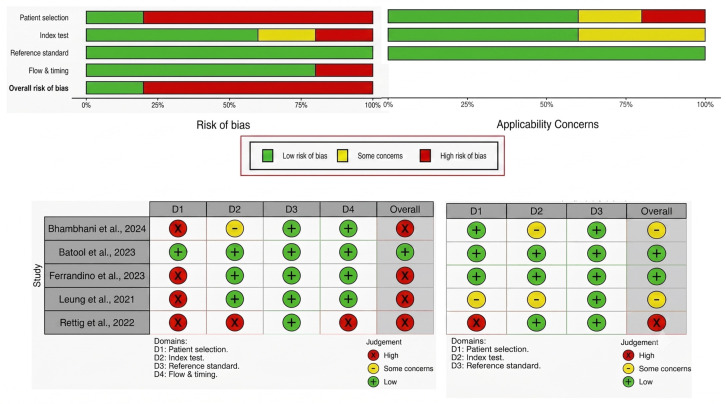
Risk of bias and applicability concerns summary (QUADAS-2). The studies evaluated in this risk of bias analysis are Bhambhani et al. [23], Batool et al. [21], Ferrandino et al. [22], Leung et al. [25], and Rettig et al. [20]. Created using robvis web application (available at: https://www.riskofbias.info/welcome/robvis-visualization-tool, accessed on 17 April 2026). Note: Gunning et al. (2023) [19] was excluded from this assessment as it is an analytical validation study without a clinical cohort.

The GRADE assessment for this quantitative synthesis indicated a moderate certainty of evidence regarding the cross-sectional diagnostic accuracy of cfHPV-DNA fragmentomic assays. Confidence in the pooled estimates was downgraded by one level due to a moderate risk of bias identified in the QUADAS-2 assessment (Figure 3). Of note, while Gunning et al. [19] was retained in the qualitative synthesis because it provides the analytical validation of the NavDx^®^ (Naveris, Waltham, MA, USA) assay used by several included clinical studies, it was excluded from the QUADAS-2 risk of bias assessment because it constitutes an analytical validation study rather than a diagnostic accuracy study with a consecutive clinical cohort. This risk primarily stems from non-consecutive patient selection (e.g., retrospective case–control designs) in a subset of the included cohorts. However, there was no serious evidence of inconsistency or indirectness. Despite the intentional methodological diversity explored in this review, specificity metrics remained universally robust (~100%), and sensitivity was consistently high across plasma samples from symptomatic patients. While a minor concern for imprecision remains due to the relatively small sample sizes, these factors do not preclude firm clinical conclusions regarding rule-in diagnostic validity in HPV-driven malignancies (Supplementary Table S5).

## 4. Discussion

### 4.1. Summary of Main Results

This systematic review and meta-analysis support the emerging consensus that fragmentomics-based cfHPV-DNA constitutes a highly promising, non-invasive biomarker for HPV-driven malignancies. The observed diagnostic performance (pooled DOR of 205.73 (95% CI: 44.72–946.38)) underscores the considerable discriminative capacity of viral liquid biopsy for detecting established disease. Although most included cohorts enrolled patients with OPSCC, the successful inclusion and detection of cervical cancer cases [25] provides a preliminary proof-of-concept that cfHPV-DNA fragmentomics may have translational relevance beyond the oropharynx, although the scarcity of gynecologic data demands rigorous validation. Methodologically, uniform use of plasma rather than serum likely contributes to assay fidelity by minimizing leukocyte-derived genomic DNA dilution during clotting, thereby preserving the endogenous fragment-length profile [28].

Biologically, tumor-derived cfHPV-DNA displays a distinct short-fragment signature (viral median ≈ 146 bp) relative to background host cfDNA (≈167 bp) when profiled within the same samples, supporting viral fragmentomics as an enrichment strategy for tumor-derived DNA [25]. Consistent with these properties, conventional ~77 bp amplicons were sufficient for reliable plasma detection, whereas physiological transrenal filtration necessitated ultrashort targets (<50 bp) to achieve sensitive detection in urine, highlighting the matrix-dependent nature of assay design [23]. Clinically, cfHPV-DNA assays demonstrated high sensitivity (>90%) for macroscopic disease in diagnostic OPSCC cohorts [22], whereas sensitivity declined in pre-diagnostic settings (e.g., ~30–43% up to 19–43 months before clinical presentation), consistent with a tumor-burden threshold for reliable shedding [20]. In contrast, specificity remained high (~100%) across symptomatic and asymptomatic populations in these series.

### 4.2. Results in the Context of Published Literature

In the broader landscape of virus-independent malignancies, liquid biopsy has increasingly pivoted toward complex fragmentomic profiling. Because discriminating tumor-derived cfDNA from the massive background of hematopoietic wild-type DNA is inherently challenging, current state-of-the-art assays, like those developed for colorectal or breast cancers, rely on multi-omics integration. This approach fuses fragmentomic features (size, end-motifs) with epigenomic layers (methylation, nucleosome positioning) to stabilize weak signals and reduce false positives [29,30,31,32]. In this context, HPV-driven oncology enjoys a distinct biological advantage: the viral genome represents a non-self, pathognomonic target. This distinct “foreignness” provides a superior signal-to-noise ratio, offering a streamlined avenue for detection that is often more analytically accessible than the deconvolution of host-derived signals [33].

To illustrate the potential value of this overlooked compartment, studies of virus-independent solid tumors have demonstrated the diagnostic utility of host-derived fragmentomic biomarkers. In colorectal cancer, genome-wide cfDNA fragmentation profiling approaches based on whole-genome sequencing and machine-learning integration of multiple fragmentation features have achieved sensitivities approaching 89–95% with specificities of 96–98%, including robust performance in early-stage disease [29,31]. These models leverage host-derived structural characteristics such as fragment length distributions, genome-wide fragmentation profiles, end-motif signatures, and nucleosome positioning patterns to generate highly discriminative cancer signatures. More recently, the fragmentomics literature has increasingly emphasized the integration of multiple fragmentomic features and complementary molecular modalities to further improve cancer detection and tissue-of-origin inference [32]. The absence of comparable investigations in HPV-driven malignancies therefore represents a significant knowledge gap and an important opportunity for future research.

However, our synthesis delineates a critical biological boundary to this advantage, namely, the dependency on tumor burden. While the viral target resolves the issue of specificity (non-self), it cannot entirely overcome the physics of shedding thresholds. This limitation is clinically corroborated by Rettig et al., who identified a stark dichotomy in pre-treatment detection rates based on nodal status: while TTMV-HPV DNA was detectable in 95% of patients with clinical N1-N3 disease, detection rates plummeted to 36% in patients with N0 disease [34]. Validating this volumetric dependency, Cooke et al. recently demonstrated that viral fragment levels scale linearly with aggregate metabolic tumor volume on PET/CT, reporting significantly increased odds of high fragment shedding in advanced cT and cN stages compared to early-stage disease [35].

This limitation is particularly relevant when considering cervical cancer, which remains markedly underrepresented in the fragmentomic literature despite being the most prevalent HPV-driven malignancy worldwide. Importantly, the absence of cervical cancer fragmentomic studies should not be interpreted as a lack of rationale for liquid biopsy in gynecological oncology. Indeed, previous studies have demonstrated that circulating HPV DNA may serve as a biomarker of treatment response and disease monitoring in locally advanced cervical cancer [36]. However, these investigations have primarily focused on viral DNA detection and quantification rather than structural fragmentation characteristics. Consequently, whether the fragment size profiles, shedding dynamics, and analytical strategies currently described in OPSCC can be directly translated to cervical cancer remains unknown. This represents a significant knowledge gap and highlights the need for dedicated prospective studies evaluating cfDNA fragmentation patterns in gynecological HPV-driven malignancies.

This dependency has profound implications for primary diagnosis, as compromised assay sensitivity in N0 disease or low-volume lesions creates “blind intervals” where malignancy evades detection. This clinical vulnerability was starkly illustrated by the early termination of a prospective de-escalation trial at Memorial Sloan Kettering (MSK); in this study, post-operative TTMV-HPV DNA negativity failed to predict certain clinical recurrences, directly mirroring the sensitivity gaps observed in early-stage diagnostic cohorts [37]. Consequently, while TTMV-HPV DNA is an effective tool for identifying macroscopic disease, a negative result in an asymptomatic individual indicates a sub-threshold tumor burden rather than the definitive absence of microscopic carcinogenesis.

Therefore, while the specific targeting of TTMV-HPV DNA offers a strong primary axis for diagnosis, the field is increasingly recognizing the necessity of multimodal integration to close this sensitivity gap. As emphasized by Lu et al., combining viral detection with host-derived multi-omics fragmentomics allows for the recovery of pan-tumor signals (via methylation or copy number variations) even when viral copies are beneath the limit of detection [32].

This fusion of modalities mitigates the biological heterogeneity demonstrated by Kumari et al., who found that while total cfDNA integrity is generally altered in cancer, the integrity index (ALU247/ALU115) was paradoxically higher in p16-negative cases compared to p16-positive disease (*p* ≈ 0.05), confirming that host fragmentation patterns are complex and require multiparametric stabilization to be useful in a diagnostic setting [38].

From a health economics perspective, despite these biological nuances, the viral-targeted approach remains the most viable entry point for clinical workup. By focusing on a targeted viral signature, TTMV-based assays avoid the prohibitive costs of comprehensive multi-omics panels in the first-line setting. Ward et al. simulated the economic impact of this strategy, estimating that while molecular testing initially costs more than standard workups ($20,756 vs. $11,674 over 5 years in a surveillance model), this difference serves as a proxy for the potential savings in a diagnostic pathway by reducing futile imaging for benign mimics. Thus, the current evidence supports a paradigm where viral fragmentomics serves as the cost-effective backbone of diagnosis, potentially supplemented by multi-omics integration only when high clinical suspicion persists despite a negative viral test [39].

### 4.3. Strengths and Weaknesses

The primary strength of this systematic review and meta-analysis lies in its stringent methodological framework and its identification of critical knowledge gaps within the field. By strictly adhering to PRISMA guidelines and conducting comprehensive QUADAS-2 and GRADE assessments, we provide a transparent evaluation of the current evidence base. Crucially, this review explicitly exposes a significant hiatus in the literature regarding HPV-driven malignancies, particularly cervical cancer. Although host-derived fragmentomics is heavily researched in virus-independent solid tumors, our systematic search formally revealed that its specific application as a diagnostic biomarker in HPV-driven malignancies remains largely uninvestigated. Furthermore, while most of the existing research has prioritized longitudinal monitoring and prognostication using binary viral detection (presence/absence) [36], our work specifically isolates the diagnostic value of fragmentation pattern analysis. By distinguishing the quantification of viral load from the characterization of fragment size profiles, we highlight the unmet need for fragmentomic validation in gynecological malignancies, which have been underrepresented compared to oropharyngeal models. Moreover, our strict focus on pre-analytical variables, emphasizing the biological necessity of utilizing plasma over serum to preserve the endogenous cfDNA fragmentomic profile, strengthens the internal validity of our quantitative synthesis. Clinically, this review bridges a critical gap by not only calculating aggregated diagnostic metrics but also contextualizing these findings within the biological constraints of tumor-burden shedding thresholds, thereby delineating the assay’s utility in diagnostic versus screening scenarios.

However, several limitations must be acknowledged, reflecting both the constraints of this review and the nascent state of the viral fragmentomics field. First, the limited number of eligible cohorts (fewer than 10 studies) restricted our statistical power to formally assess publication bias. In strict accordance with Cochrane guidelines, asymmetry tests such as Deeks’ funnel plot were precluded to avoid misleading statistical artifacts. Second, the current literature is heavily reliant on retrospective, case–control study designs. As captured by our QUADAS-2 assessment, the inclusion of patients with known, established malignancies introduces inherent ascertainment bias, which likely overestimates the diagnostic sensitivity compared to what would be observed in a true prospective study.

Furthermore, the biological scope of the available data remains uneven. The predominant focus on head and neck cancers limits the immediate generalizability of our findings to cervical cancer or other HPV-related cancers, where fragmentomic dynamics may differ due to anatomical and physiological clearance mechanisms. Additionally, technical heterogeneity across studies, ranging from algorithmic ddPCR to high-resolution NGS, remains a challenge. While our random-effects meta-analysis of the diagnostic odds ratio (DOR) accounts for this variance, the lack of standardized pre-analytical pipelines across the industry represents a persistent hurdle for universal clinical implementation.

Finally, we must acknowledge that, although cfDNA fragmentation patterns represent a biologically attractive biomarker strategy, the current evidence base remains limited. Most available studies were not specifically designed to compare fragmentation-based approaches with conventional HPV DNA detection assays. Consequently, it remains unclear whether fragmentation profiling provides clinically meaningful incremental value beyond sensitive viral DNA detection alone. Dedicated head-to-head comparative studies are required to determine whether these structural biomarkers provide clinically meaningful improvements in sensitivity, specificity, or early-stage detection beyond conventional HPV DNA assays, particularly in low-tumor-burden disease.

### 4.4. Implications for Practice and Future Research

The findings of this quantitative synthesis delineate a clear roadmap for the clinical translation of viral fragmentomics. Given the identified “tumor-burden threshold” for reliable detection, the immediate clinical utility of these assays lies in risk-stratified surveillance rather than unselected population screening. For HPV-driven head and neck squamous cell carcinoma (HNSCC), where incidence is rising and occult recurrence remains a clinical challenge, practice should shift toward implementing serial cfHPV-DNA testing in high-risk cohorts. In this setting, the assay’s dynamic range allows for the detection of molecular recurrence months before radiological manifestation [21]. However, to mitigate the risk of false negatives in low-volume disease, clinical protocols should likely adopt a “viral-first” approach: utilizing the high-specificity TTMV signal as the primary trigger for investigation, while retaining a low threshold for anatomical imaging in symptomatic patients with negative liquid biopsies.

To address the biological constraints of viral shedding in early-stage or microscopic disease, future assay development should follow a tiered optimization strategy. The first logical step involves maximizing the diagnostic yield of the viral target itself through multiparametric fragmentomics. Rather than relying solely on fragment size, future protocols should integrate viral end-motif profiling, jagged-end analysis, and topological features into a single cohesive viral signature to refine detection sensitivity without significantly escalating costs [40]. However, should these optimized viral metrics remain constrained by the absolute physical limit of shedding in microscopic lesions, the strategy must then pivot toward integrated multi-omics. By layering host-derived epigenetic signatures, such as methylation profiling or chromatin accessibility scores, alongside the viral signal, next-generation assays could capture the host’s molecular response to the tumor (a “pan-tumor” signal) even when viral copies are beneath the limit of detection, thus enabling true primary screening in asymptomatic populations [41].

Finally, the translation of these assays from research tools to universal clinical guidelines requires a rigorous overhaul of study design and standardization. The current heterogeneity in pre-analytical and analytical workflows hinders inter-institutional data comparability. To address this, the field must align with ongoing collaborative initiatives such as the Blood Profiling Atlas in Cancer (BLOODPAC) Consortium [42]. Adopting consensus protocols for pre-analytical handling, including standardized blood collection, stabilization, processing times, and storage conditions, alongside the use of reference materials and common bioinformatics pipelines is essential to ensure reproducibility. Consequently, future evidence must stem from large-scale prospective longitudinal cohorts that rigorously adhere to these standardized frameworks. Crucially, these studies must be powered to evaluate distinct HPV-driven populations independently, explicitly comparing the fragmentomic kinetics of HNSCC versus gynecological malignancies. Validating whether the ultrashort viral signature is conserved across these anatomical sites, where clearance mechanisms differ, is a priority to define anatomically adjusted diagnostic thresholds and unlock the full pan-tumor potential of this biomarker.

## 5. Conclusions

In conclusion, this systematic review and meta-analysis highlight the promising diagnostic potential of cfHPV-DNA fragmentation pattern-based assays for HPV-driven malignancies. While current assays leverage a characteristic ultrashort viral signature to discriminate tumor-derived signals from background cfDNA, it remains to be definitively proven whether fragment sizing strictly outperforms binary viral detection in clinical practice.

Our findings show that while this approach yields high sensitivity for detecting established, macroscopic disease, its performance in asymptomatic or microscopic settings remains severely constrained by biological shedding thresholds.

Consequently, while current evidence supports the analytical validity of viral fragmentomics for rule-in diagnostic applications, its translation into a universal clinical standard requires confirmation through large-scale, prospective longitudinal studies. Such studies must prioritize the standardization of pre-analytical protocols and define anatomically specific diagnostic thresholds across diverse HPV-driven malignancies. Furthermore, future research must determine whether these fragmentation patterns correlate with disease severity. Ultimately, while current methodologies exploit the analytical simplicity of viral targets, host-derived fragmentomics remains a critical, overlooked compartment that warrants thorough exploration to capture the full scope of underlying tumor dynamics.

## Data Availability

No new data were created or analyzed in this study. Data sharing is not applicable to this article as all data were obtained from previously published studies, which are cited within the manuscript.

## Supplementary Materials

The following supporting information can be downloaded at https://www.mdpi.com/article/10.3390/ijms27146372/s1.

## Abbreviations

The following abbreviations are used in this manuscript:

AJCC	American Joint Committee on Cancer
AUC	Area Under the Curve
bp	Base Pair
cfDNA	Cell-Free DNA
cfHPV-DNA	Cell-Free Human Papillomavirus DNA
CHAMP-16	Circular-HPV-Associated Molecular Pattern (16-target ultrashort ddPCR assay)
CI	Confidence Interval
CIN	Cervical Intraepithelial Neoplasia
ctDNA	Circulating Tumor DNA
CTCs	Circulating Tumor Cells
DOR	Diagnostic Odds Ratio
ddPCR	Droplet Digital Polymerase Chain Reaction
DTA	Diagnostic Test Accuracy
EDTA	Ethylenediaminetetraacetic Acid
FIGO	International Federation of Gynecology and Obstetrics
FN	False Negative
FP	False Positive
HNSCC	Head and Neck Squamous Cell Carcinoma
HPV	Human Papillomavirus
HSROC	Hierarchical Summary Receiver Operating Characteristic
IHC	Immunohistochemistry
IQR	Interquartile Range
NGS	Next-Generation Sequencing
OPSCC	Oropharyngeal Squamous Cell Carcinoma
PCR	Polymerase Chain Reaction
PRISMA	Preferred Reporting Items for Systematic Reviews and Meta-Analyses
QUADAS-2	Quality Assessment of Diagnostic Accuracy Studies–2
ROC	Receiver Operating Characteristic
SROC	Summary Receiver Operating Characteristic
SE	Standard Error
SPEC	Specificity
SENS	Sensitivity
TN	True Negative
TP	True Positive
TR-ctDNA	Transrenal Circulating Tumor DNA
TTMV-HPV DNA	Tumor Tissue–Modified Viral HPV DNA (NavDx®, Naveris, Waltham, MA, USA)
WGS	Whole-Genome Sequencing
