# Supplementary material for: Cell-Free DNA Fragmentation Patterns as Biomarkers for Human Papillomavirus-Related Cancers: A Systematic Review of Methodological Diversity and Diagnostic Performance
Source: Int J Mol Sci. 2026 Jul 17;27(14):6372. doi: 10.3390/ijms27146372 (PMC13411076; doi:10.3390/ijms27146372)
Supplement: Supplementary file 1 [file ijms-27-06372-s001.zip › ijms-4422600-supplementary.pdf]

Table S1. PRISMA 2020 Checklist

Section and Topic	Item #	Checklist item	Location where item is reported
**TITLE**			
Title	1	Identify the report as a systematic review.	p.1, lines 1–4
**ABSTRACT**			
Abstract	2	See the PRISMA 2020 for Abstracts checklist.	p.1–2, lines 28–47
**INTRODUCTION**			
Rationale	3	Describe the rationale for the review in the context of existing knowledge.	p.2–3, lines 52–111
Objectives	4	Provide an explicit statement of the objective(s) or question(s) the review addresses.	p.3, lines 112–116
**METHODS**			
Eligibility criteria	5	Specify the inclusion and exclusion criteria for the review and how studies were grouped for the syntheses.	Section 2.2, p.3, lines 127–134; Table S3
Information sources	6	Specify all databases, registers, websites, organisations, reference lists and other sources searched or consulted to identify studies. Specify the date when each source was last searched or consulted.	Section 2.3, p.3-4, lines 136–145
Search strategy	7	Present the full search strategies for all databases, registers and websites, including any filters and limits used.	Section 2.3, p.3-4, lines 136–145; Table S2 (Search Strategy)
Selection process	8	Specify the methods used to decide whether a study met the inclusion criteria of the review, including how many reviewers screened each record and each report retrieved, whether they worked independently, and if applicable, details of automation tools used in the process.	Section 2.4, p.4, lines 147–157
Data collection process	9	Specify the methods used to collect data from reports, including how many reviewers collected data from each report, whether they worked independently, any processes for obtaining or confirming data from study investigators, and if applicable, details of automation tools used in the process.	Section 2.4, p.4, lines 147–157
Data items	10a	List and define all outcomes for which data were sought. Specify whether all results that were compatible with each outcome domain in each study were sought (e.g. for all measures, time points, analyses), and if not, the methods used to decide which results to collect.	Section 2.4, p.4, lines 147–157
	10b	List and define all other variables for which data were sought (e.g. participant and intervention characteristics, funding sources). Describe any assumptions made about any missing or unclear information.	Section 2.4, p.4, lines 147–157
Study risk of bias assessment	11	Specify the methods used to assess risk of bias in the included studies, including details of the tool(s) used, how many reviewers assessed each study and whether they worked independently, and if applicable, details of automation tools used in the process.	Section 2.5, p.4, lines 159–162

Section and Topic	Item #	Checklist item	Location where item is reported
Effect measures	12	Specify for each outcome the effect measure(s) (e.g. risk ratio, mean difference) used in the synthesis or presentation of results.	Section 2.6, p.4, lines 163–172
Synthesis methods	13a	Describe the processes used to decide which studies were eligible for each synthesis (e.g. tabulating the study intervention characteristics and comparing against the planned groups for each synthesis (item #5)).	Sections 2.2 and 2.6, p.3–4, lines 127–134 and 164–178
	13b	Describe any methods required to prepare the data for presentation or synthesis, such as handling of missing summary statistics, or data conversions.	Section 2.6 Statistical Analysis, p.4, lines 164–178
	13c	Describe any methods used to tabulate or visually display results of individual studies and syntheses.	Figure 1; Figure 2; Figure 3; Tables 1–2
	13d	Describe any methods used to synthesize results and provide a rationale for the choice(s). If meta-analysis was performed, describe the model(s), method(s) to identify the presence and extent of statistical heterogeneity, and software package(s) used.	Section 2.6 p.4, lines 164–178
	13e	Describe any methods used to explore possible causes of heterogeneity among study results (e.g. subgroup analysis, meta-regression).	Not performed due to limited number of studies
	13f	Describe any sensitivity analyses conducted to assess robustness of the synthesized results.	Not performed
Reporting bias assessment	14	Describe any methods used to assess risk of bias due to missing results in a synthesis (arising from reporting biases).	Section 2.6, p.4, lines 164–178
Certainty assessment	15	Describe any methods used to assess certainty (or confidence) in the body of evidence for an outcome.	Section 2.5, p.4, lines 160–162; Section 3.4, p. 10, lines 291–307; Table S5
**RESULTS**			
Study selection	16a	Describe the results of the search and selection process, from the number of records identified in the search to the number of studies included in the review, ideally using a flow diagram.	Section 3.1 p.4–5, lines 180–204; Figure 1
	16b	Cite studies that might appear to meet the inclusion criteria, but which were excluded, and explain why they were excluded.	Section 3.1 p.4–5, lines 180–204
Study characteristics	17	Cite each included study and present its characteristics.	Section 3.2, pp.5–8, lines 206–234; Tables

Section and Topic	Item #	Checklist item	Location where item is reported
			1–2 and S4
Risk of bias in studies	18	Present assessments of risk of bias for each included study.	Section 3.4, p.10–11, lines 292–348; Figure 3
Results of individual studies	19	For all outcomes, present, for each study: (a) summary statistics for each group (where appropriate) and (b) an effect estimate and its precision (e.g. confidence/credible interval), ideally using structured tables or plots.	Table 2, pp.8–9; Section 3.2, lines 206–234
Results of syntheses	20a	For each synthesis, briefly summarise the characteristics and risk of bias among contributing studies.	Sections 3.3–3.4, pp.9–10, lines 252–308
	20b	Present results of all statistical syntheses conducted. If meta-analysis was done, present for each the summary estimate and its precision (e.g. confidence/credible interval) and measures of statistical heterogeneity. If comparing groups, describe the direction of the effect.	Section 3.3 p.9, lines 252–290; Figure 2
	20c	Present results of all investigations of possible causes of heterogeneity among study results.	Section 3.3, lines 252–290
	20d	Present results of all sensitivity analyses conducted to assess the robustness of the synthesized results.	Not performed
Reporting biases	21	Present assessments of risk of bias due to missing results (arising from reporting biases) for each synthesis assessed.	Section 3.4, p.10, lines 292–348. Figure 3
Certainty of evidence	22	Present assessments of certainty (or confidence) in the body of evidence for each outcome assessed.	Section 3.4, p.10, lines 292–348; Table S5
**DISCUSSION**			
Discussion	23a	Provide a general interpretation of the results in the context of other evidence.	Section 4.1–4.2, pp.11–12, lines 351–456
	23b	Discuss any limitations of the evidence included in the review.	Section 4.3, pp.13, lines 458–506
	23c	Discuss any limitations of the review processes used.	Section 4.3, pp.13, lines 458–506
	23d	Discuss implications of the results for practice, policy, and future research.	Section 4.4, pp.13–14, lines

Section and Topic	Item #	Checklist item	Location where item is reported
			508–548
OTHER INFORMATION			
Registration and protocol	24a	Provide registration information for the review, including register name and registration number, or state that the review was not registered.	Section 2.1, p.3, lines 120–125
	24b	Indicate where the review protocol can be accessed, or state that a protocol was not prepared.	Section 2.1, p.3, lines 120–125; Supplementary Materials: Table S1 (PRISMA Checklist) and Table S2 (Search Documentation)
	24c	Describe and explain any amendments to information provided at registration or in the protocol.	No amendments to protocol were made after registration
Support	25	Describe sources of financial or non-financial support for the review, and the role of the funders or sponsors in the review.	Funding Statement, p.16, line 582
Competing interests	26	Declare any competing interests of review authors.	Conflicts of Interest, p.16, line 589
Availability of data, code and other materials	27	Report which of the following are publicly available and where they can be found: template data collection forms; data extracted from included studies; data used for all analyses; analytic code; any other materials used in the review.	Data Availability Statement, p.16, lines 585–587

From: Page MJ, McKenzie JE, Bossuyt PM, Boutron I, Hoffmann TC, Mulrow CD, et al. The PRISMA 2020 statement: an updated guideline for reporting systematic reviews. BMJ 2021;372:n71. doi: 10.1136/bmj.n71. This work is licensed under CC BY 4.0. To view a copy of this license, visit https://creativecommons.org/licenses/by/4.0/

Table S2. Full search strategy for each database. (at 09.02.2026)

Database	Topic	Query Syntax
PubMed	Host cfDNA Integrity	("Cell-Free Nucleic Acids"[Mesh] OR "cell-free DNA"[Title/Abstract] OR "cfDNA"[Title/Abstract] OR "circulating DNA"[Title/Abstract] OR "plasma DNA"[Title/Abstract] OR "serum DNA"[Title/Abstract] OR "liquid biopsy"[Title/Abstract]) AND ("integrity"[Title/Abstract] OR "fragmentation"[Title/Abstract] OR "fragmentomics"[Title/Abstract] OR "size"[Title/Abstract] OR "length"[Title/Abstract] OR "Alu"[Title/Abstract] OR "Alu115"[Title/Abstract] OR "Alu247"[Title/Abstract] OR "LINE-1"[Title/Abstract] OR "DNA index"[Title/Abstract] OR "long fragment*"[Title/Abstract] OR "short fragment*"[Title/Abstract]) AND ("Uterine Cervical Neoplasms"[Mesh] OR "Head and Neck Neoplasms"[Mesh] OR "Anus Neoplasms"[Mesh] OR "Vulvar Neoplasms"[Mesh] OR "Penile Neoplasms"[Mesh] OR "Vaginal Neoplasms"[Mesh] OR "cervical cancer"[Title/Abstract] OR "cervix cancer"[Title/Abstract] OR "oropharyngeal cancer"[Title/Abstract] OR "anal cancer"[Title/Abstract] OR "head and neck cancer"[Title/Abstract] OR "vulvar cancer"[Title/Abstract] OR "penile cancer"[Title/Abstract] OR "vagina cancer"[Title/Abstract] OR "HPV-related"[Title/Abstract])
	Viral HPV-DNA Fragmentomics	("Cell-Free Nucleic Acids"[Mesh] OR "cell-free DNA"[Title/Abstract] OR "cfDNA"[Title/Abstract] OR "ctDNA"[Title/Abstract] OR "circulating tumor DNA"[Title/Abstract] OR "liquid biopsy"[Title/Abstract]) AND ("Papillomaviridae"[Mesh] OR "HPV"[Title/Abstract] OR "Human Papillomavirus"[Title/Abstract]) AND ("fragmentomics"[Title/Abstract] OR "fragmentation profile"[Title/Abstract] OR "fragment size"[Title/Abstract] OR "read length"[Title/Abstract] OR "size profile"[Title/Abstract] OR "TTMV"[Title/Abstract] OR "tumor tissue modified"[Title/Abstract] OR "integration"[Title/Abstract] OR "viral integration"[Title/Abstract] OR "next-generation sequencing"[Title/Abstract] OR "NGS"[Title/Abstract] OR "deep sequencing"[Title/Abstract]) AND ("Uterine Cervical Neoplasms"[Mesh] OR "Head and Neck Neoplasms"[Mesh] OR "Anus Neoplasms"[Mesh] OR "Vulvar Neoplasms"[Mesh] OR "Penile Neoplasms"[Mesh] OR "cervical cancer"[Title/Abstract] OR "oropharyngeal cancer"[Title/Abstract] OR "anal cancer"[Title/Abstract])
Embase	Host cfDNA Integrity	('cell free deoxyribonucleic acid'/exp OR 'circulating tumor deoxyribonucleic acid'/exp OR 'liquid biopsy'/exp OR 'cell free dna':ti,ab,kw OR 'cfdna':ti,ab,kw OR 'circulating dna':ti,ab,kw OR 'plasma dna':ti,ab,kw OR 'serum dna':ti,ab,kw) AND ('dna fragmentation'/exp OR 'integrity index':ti,ab,kw OR 'integrity ratio':ti,ab,kw OR 'fragmentation index':ti,ab,kw OR 'alu repeat':ti,ab,kw OR 'alu element':ti,ab,kw OR 'alu sequence':ti,ab,kw OR 'alu115':ti,ab,kw OR 'alu247':ti,ab,kw OR 'alu ratio':ti,ab,kw OR 'line-1':ti,ab,kw OR 'dna index':ti,ab,kw OR 'fragment size':ti,ab,kw OR 'dna size':ti,ab,kw OR 'long fragment':ti,ab,kw OR 'short fragment':ti,ab,kw) AND ('uterine cervix cancer'/exp OR 'head and neck cancer'/exp OR 'anus cancer'/exp OR 'vulva cancer'/exp OR 'penis cancer'/exp OR 'vagina cancer'/exp OR 'cervical cancer':ti,ab,kw OR 'oropharyngeal cancer':ti,ab,kw OR 'anal cancer':ti,ab,kw OR 'head and neck cancer':ti,ab,kw OR 'vulvar cancer':ti,ab,kw OR 'penile cancer':ti,ab,kw OR 'vaginal cancer':ti,ab,kw OR 'hpv related':ti,ab,kw)
	Viral HPV-DNA Fragmentomics	(human papillomavirus'/exp OR 'human papillomavirus' OR hpv:ti,ab,kw OR 'hpv dna':ti,ab,kw OR 'circulating hpv dna':ti,ab,kw OR 'viral dna':ti,ab,kw)AND ('cell free deoxyribonucleic acid'/exp OR 'circulating tumor deoxyribonucleic acid'/exp OR 'liquid biopsy'/exp OR 'cell free dna':ti,ab,kw OR cfdna:ti,ab,kw OR ctdna:ti,ab,kw OR 'circulating dna':ti,ab,kw OR 'plasma dna':ti,ab,kw OR 'serum dna':ti,ab,kw OR 'circulating hpv dna':ti,ab,kw) AND ('dna fragmentation'/exp OR 'dna fragmentation':ti,ab,kw OR 'dna integrity':ti,ab,kw OR'integrity index':ti,ab,kw OR 'integrity ratio':ti,ab,kw OR 'fragmentation index':ti,ab,kw OR 'fragment size':ti,ab,kw OR 'fragment length':ti,ab,kw OR 'fragmentation pattern*':ti,ab,kw OR 'short fragment':ti,ab,kw OR 'long fragment':ti,ab,kw OR 'viral fragment size':ti,ab,kw OR 'hpv fragment size':ti,ab,kw) AND ('uterine cervix cancer'/exp OR 'head and neck cancer'/exp OR 'anus cancer'/exp OR 'vulva cancer'/exp OR 'penis cancer'/exp OR 'vagina cancer'/exp OR 'cervical cancer':ti,ab,kw OR 'oropharyngeal cancer':ti,ab,kw OR 'anal cancer':ti,ab,kw OR 'head and neck cancer':ti,ab,kw OR 'vulvar cancer':ti,ab,kw OR 'penile cancer':ti,ab,kw OR 'vaginal cancer':ti,ab,kw OR 'hpv related':ti,ab,kw OR 'hpv-associated':ti,ab,kw)
Cochrane Library	All	#1 MeSH descriptor: [Papillomaviridae] explode all trees; #2 (HPV OR "Human Papillomavirus" OR "cervical cancer" OR "oropharyngeal cancer" OR "anal cancer" OR "head and neck cancer" OR "vulvar cancer" OR "vaginal cancer" OR "penile cancer"):ti,ab,kw; #3 MeSH descriptor: [Cell-Free Nucleic Acids] explode all trees; #4 ("cell-free DNA" OR cfDNA OR ctDNA OR "circulating tumor DNA" OR "liquid biopsy" OR "plasma DNA" OR "serum DNA"):ti,ab,kw; #5 ("fragmentation" OR "fragmentomics" OR "integrity" OR "size" OR "length" OR "Alu" OR "LINE-1" OR "TTMV" OR "viral integration" OR "end-motif"):ti,ab,kw; #6 #1 OR #2; #7 #3 OR #4; #8 #6 AND #7 AND #5.
ClinicalTrials.gov	All	Condition/disease: HPV Cancer OR Cervical Cancer OR Oropharyngeal Cancer. Other terms: "cell-free DNA" OR fragmentomics OR TTMV
Web of Science	All	(("HPV" OR "Human Papillomavirus" OR "cervical cancer" OR "oropharyngeal cancer" OR "anal cancer" OR "head and neck cancer") AND ("cell-free DNA" OR "cfDNA" OR "ctDNA" OR "liquid biopsy") AND ("fragmentation" OR "fragmentomics" OR "integrity" OR "Alu" OR "LINE-1" OR "TTMV" OR "size profile" OR "fragment size"))
WHO ICTRP	All	"HPV cell-free DNA" OR "HPV liquid biopsy" OR "HPV fragmentomics" OR "TTMV HPV"
EU Clinical Trials	All	Search 1: HPV AND "cell-free DNA"; Search 2: HPV AND fragmentomics; Search 3: HPV AND "liquid biopsy"

Grey literature rationale: Additionally, annual conference abstracts from major oncology and virology congresses (e.g., ASCO, ESMO, IPVC) were screened to minimize publication bias. Semantic Scholar was used as a supplementary search tool to identify additional relevant literature, ensuring broader coverage of grey literature and studies not captured through traditional bibliographic databases. A manual search of the reference lists of included articles was carried out to identify potential additional studies. If the same study was reported in different articles, the one with the largest sample size or presenting data more suitable for our specific aim was selected. This strategy was implemented to ensure the identification of: (1) recent high-impact studies presented at major conferences but not yet peer-reviewed (mitigating time-lag bias); (2) studies with negative or inconclusive results that are often underrepresented in major journals (mitigating publication bias); and (3) relevant studies indexed in non-medical databases using AI-driven semantic discovery.

Table S3. Inclusion and exclusion criteria based on PECO

Rule	Inclusion Criteria	Exclusion Criteria
Study Type	Original research or replication studies published in peer-reviewed journals.<br>No limitations imposed regarding publication date.	In vitro or animal research studies without human data, case reports, review articles, editorials and perspectives.
Participants	Patients with histopathologically confirmed HPV-driven malignancies (any stage I–IV).<br>High-grade precursor lesions (e.g., HSIL).	Pregnant women (due to confounding fetal cfDNA fragmentation).<br>Patients undergoing active chemo/radiotherapy at sampling.<br>History of synchronous non-HPV malignancies.<br>Only animal models.<br>Only cell lines.
Exposure	Assays evaluating physical properties of cfDNA (size, integrity, fragmentation).<br>Matrices: Plasma or Serum.<br>Methods: qPCR, dPCR, NGS.	Studies measuring solely total cfDNA concentration.<br>Studies measuring solely absolute viral load (copies/mL) without integrity metrics.<br>Methylation assays without fragmentation data.
Comparators	Healthy volunteers.<br>Benign/inflammatory conditions in corresponding anatomical sites.<br>HPV-negative cancers	Absence of a control group.<br>Comparison of technical isolation methods without clinical diagnostic data.
Outcomes	Quantitative diagnostic accuracy metrics (TP, FP, FN, TN).<br>Sensitivity, Specificity, AUC.<br>Differential fragmentation indices (e.g., mean fragment size).	Studies reporting only qualitative presence/absence without accuracy data.<br>Studies focusing solely on prognosis/survival without a diagnostic baseline comparison.

Table S4. Technical specifications and assay characteristics of the included cfHPV-DNA fragmentomics studies.

Study (Author/ Year)	Matrix	Blood Tube	Processing Time	Centrifugation	DNA Extraction Kit	Assay Platform	Target Region	Method of size selection	Cut-off
Gunning et al., 2023[19][1]	Plasma	Cell-Free DNA BCT® Streck	Validated up to 7 days	Double Spin (Standard protocol)[2]	QIAamp Circulating Nucleic Acid Kit (Qiagen®)	ddPCR (NavDx®)[3]	TTMV–HPV DNA (HPV16, 18, 31, 33, 35)	Algorithmic fragment-size profiling (K-means clustering/Gaussian mixture model)	Positive score (>0 fragments/mL)
Bhambhani et al., 2024[23]	Plasma (matched with Urine)	Urine: EDTA; Plasma: Cell-Free DNA BCT® Streck	< 4h	Urine: Single Spin / Plasma: Double Spin (Standard)	Urine: Q Sepharose resin-based / Plasma: QIAamp-Qiagen® (Standard)	Ultrashort ddPCR (CHAMP-16 Assay)	HPV16 (E6 gene) (Ultrashort amplicons)	Physical (Beads + Resin)	Presence of Ultrashort HPV16 fragments (>0 copies)
Batool et al., 2023[21]	Plasma	Cell-Free DNA BCT® Streck	≤ 7 days (per NavDx protocol)	Double Spin (Standard Protocol)	QIAamp Circulating Nucleic Acid Kit (Qiagen®)	ddPCR (NavDx®)	TTMV – HPV DNA[1]	Primer Design / In Silico	Positive Score (>0 fragments/mL)
Ferrandino et al., 2023[22]	Plasma	Cell-Free DNA BCT® Streck	≤ 7 days (per NavDx protocol)	Double Spin (Standard Protocol)	QIAamp Circulating Nucleic Acid Kit (Qiagen®)	ddPCR (NavDx®)	TTMV – HPV DNA[1]	In silico / Primer design (Targeting short fragments)	Positive Score (>0 fragments/mL)
Leung et al., 2021[25]	Plasma	EDTA	≤ 2h	NR	QIAamp Circulating Nucleic Acid Kit (Qiagen®)	Targeted NGS (Illumina ® NextSeq/NovaSeq)	Viral-Fragmentomics (Primary: Full-length Viral Genome)	Hybrid Capture (bioinformatic size analysis)	Presence of unique HPV-mapped reads
Rettig et al., 2022[20][1]	Plasma (Archived/ Frozen)	EDTA	≤ 7 days (per NavDx protocol)	2465 g (single spin reported for biobank)	QIAamp Circulating Nucleic Acid Kit (Qiagen®)	ddPCR (NavDx®)	TTMV – HPV DNA	Algorithmic (K-Nearest Neighbors clustering of droplets based on amplicon size)	Positive Score (>0 fragments/mL)

ddPCR: digital droplet PCR; NR: not reported; TTMV : Tumor Tissue Modified Viral; [1]: NavDx uses 12 DNA biomarkers to detect and profile the fragmentation pattern of HPV DNA using droplet digital PCR; [2]: Standard Protocol refers to centrifugation at 4°C: 2000×g for 10 min, followed by 2500×g for 10 min, as validated by Gunning et al.; [3]: Excluded from quantitative meta-analysis due to pre-diagnostic design.

Table S5. GRADE Assessment of Certainty of Evidence for the Diagnostic Accuracy of Fragmentomic cfHPVDNA Assays.

Outcomes	Study Design	Risk of Bias in Individual Studies	Publication Bias	Inconsistency	Indirectness	Imprecision	Confidence in Evidence	Recommendation
Diagnostic Accuracy (Sensitivity and Specificity) of fragmentomics-based cfHPVDNA assays in HPV-driven malignancies	Prospective clinical cohorts and retrospective case-control studies.	Serious (-1)[1]	Not Serious (0)[2]	Not Serious (0)[3]	Not Serious (0)[4]	Not Serious (0)[5]	Moderate[6]	Conditional to strong recommendation for rule-in diagnostic use in clinically suspected HPV-driven malignancies; not recommended as a standalone screening tool in asymptomatic populations.

Notes:

[1] Downgraded due to a moderate risk of bias identified in QUADAS-2, primarily stemming from non-consecutive patient selection (e.g., retrospective designs) in a subset of the included cohorts.

[2] While formal statistical evaluation (e.g., Deeks' funnel plot) is underpowered with <10 studies, a comprehensive search strategy minimizes the risk of missing significant data.

[3] Despite intended methodological diversity, findings exhibit remarkable consistency: specificity is nearly 100% across all studies, and sensitivity remains consistently robust in baseline clinical cohort.

[4] Direct evidence. The studies directly assessed the intended target populations (patients presenting with clinical HPV-related cancer: OPSCC) against the appropriate gold standard (tissue p16/PCR).

[5] Although individual independent cohorts feature relatively small sample sizes, the cumulative aggregated cohort provides sufficiently narrow confidence intervals, particularly for specificity, avoiding a formal downgrade.

[6] Started at High for observational diagnostic studies, downgraded by 1 for Risk of Bias.
